# Lessons learnt recruiting to a multi-site UK cohort study to explore recovery of health and well-being after colorectal cancer (CREW study)

**DOI:** 10.1186/1471-2288-13-153

**Published:** 2013-12-28

**Authors:** Deborah Fenlon, Kim Chivers Seymour, Ikumi Okamoto, Jane Winter, Alison Richardson, Julia Addington-Hall, Jessica L Corner, Peter W Smith, Christine M May, Matthew Breckons, Claire Foster

**Affiliations:** 1 Faculty of Health Sciences, University of Southampton, Southampton SO17 1BJ, UK; 2 Southampton General Hospital, University of Southampton NHS Foundation Trust, Tremona Rd, Southampton, Hampshire SO16 6YD, UK; 3 Newcastle University, Newcastle upon Tyne, Tyne and Wear NE1 7RU, UK

**Keywords:** Cohort, Colorectal cancer, Health and wellbeing, Study processes, Recruitment

## Abstract

**Background:**

The UK leads the world in recruitment of patients to cancer clinical trials, with a six-fold increase in recruitment during 2001–2010. However, there are large variations across cancer centres. This paper details recruitment to a large multi-centre prospective cohort study and discusses lessons learnt to enhance recruitment.

**Methods:**

During CREW (ColoREctal Wellbeing) cohort study set up and recruitment, data were systematically collected on all centres that applied to participate, time from study approval to first participant recruited and the percentage of eligible patients recruited into the study.

**Results:**

30 participating NHS cancer centres were selected through an open competition via the cancer networks. Time from study approval to first participant recruited took a median 124 days (min 53, max 290). Of 1350 eligible people in the study time frame, 78% (*n* = 1056) were recruited into the study, varying from 30-100% eligible across centres. Recruitment of 1056 participants took 17 months.

**Conclusion:**

In partnership with the National Cancer Research Network, this successful study prioritised relationship building and education. Key points for effective recruitment: pre-screening and selection of centres; nurses as PIs; attendance at study days; frequent communication and a reduced level of consent to enhance uptake amongst underrepresented groups.

## Background

Recruitment of participants with cancer from clinical settings into research studies is commonly reported as slow and only a minority reach their original recruitment target [1]. The lack of agreement between expected and achieved targets for recruitment has major implications for study outcomes and the allocation of scarce resources. Studies take longer than planned and effort required to recruit hard to reach groups means they are likely to remain underrepresented, leading to bias in published research. In the United Kingdom, national support systems and infrastructure, such as the National Cancer Research Network (NCRN), part of the National Institute for Health Research (NIHR) Clinical Research Network (CRN) have been introduced with great success to increase the number of patients participating in clinical research studies, with the result that the UK leads the world in recruiting to cancer clinical trials [2]. Since the NCRN was established in 2001 recruitment rates to clinical research cancer trials have increased steadily, first quadrupling [3] and now rising to six fold, with an increase of 3.5% to 23% of all incident cancer cases [4] and 75% of studies meeting the recruitment target, compared with 39% before the NCRN was established [5]. A key feature of this success is the use of dedicated research nurses to recruit to portfolio studies. More recently, the UK government announced incentives for initiation and delivery in research in 2011 [6]. The NIHR developed the Research Support Services Framework in 2011 that required NHS service providers to publish outcomes against contract NIHR benchmarks from 2012 onwards. A key initial benchmark is that there should be 70 days or less from the time a provider of NHS services receives a valid research application to the time when that provider recruits the first patient for that study [7]. Nevertheless, there remain large variations between the ability of individual cancer centres to recruit to research. Patterson et al. [8] suggest that effective negotiation of ‘gatekeeping’ with those who mediate access to potential participants is vital to the process and outcomes of trials and the quality of evidence. They conceptualise successful recruitment as a process consisting of three phases: set up, alliance and exchange, where each phase is dependent on satisfactory negotiation of the other. We utilise their framework to describe the experience of recruiting a large cancer cohort from multiple sites across Great Britain during 2011/2012. The study successfully recruited a high percentage of eligible patients within a short time frame. The processes that were utilised to enhance recruitment are discussed, as well as difficulties encountered, and recommendations for future studies. We also present detailed data that compare individual recruiting centres including time to obtain governance approvals, time to recruit and percentage of eligible patients recruited.

## Methods

The CREW (ColoREctal Wellbeing) study is a prospective, longitudinal cohort study of 1056 colorectal cancer survivors using a mailed questionnaire survey, aiming to follow the path of recovery from colorectal cancer from baseline, prior to surgery, with regular follow-up questionnaires [9]. Despite participants having just been diagnosed with cancer we were able to recruit a very high percentage (78%) of the eligible population for this study and obtained consent for basic demographic details for a further 13%, enabling us to have basic details for 91% of the total cohort. From these data it will be possible to identify those who are at risk of making a poor or protracted recovery in order to target resource appropriately. Recruitment to the study was via NHS colorectal cancer pre-surgical clinics, with the support of dedicated research nurses. All those with a diagnosis of colorectal cancer with no distant metastases (Dukes A-C) and 18 years or over were eligible for the study. Prior diagnosis of cancer (other than non-melanomatous skin cancer or in situ carcinoma cervix) was an exclusion criterion. A particular challenge for this study was the need to minimise bias by ensuring that every individual who met these very broad criteria was approached for participation in the study and that data were collected on those who were not recruited. This required recruiting staff to provide detailed screening logs to the co-ordinating centre about numbers of people presenting who were eligible, how many were recruited, how many declined and how many were missed. Clinical trials often exclude hard to reach groups, such as the elderly and frail, however, it was necessary for us to understand the needs and recovery trajectory of this group and so it was important to include all groups in the study. It became clear that some people were electing not to join the study due to questionnaire burden, and so a second level of consent was included, whereby routinely collected data could be accessed at regular intervals and monitored over time. This constituted a second level of consent, known as reduced consent.

### Recruitment and management of the study

The study was set up and managed by the co-ordinating centre at the Macmillan Survivorship Research Group, University of Southampton. A pilot study was undertaken in 2010 in one centre to test recruitment rates and procedures. The Principal Investigator at this site was the colorectal cancer consultant nurse. Eligible patients were identified by the nurse specialist team and logged prior to primary surgery at a multi-disciplinary team (MDT) meeting. Eligible patients were informed about the study by their clinicians (either their surgeon or clinical nurse specialist) and by written information, and recruited at a pre-surgical assessment clinic by the nurse practitioner. The nurse practitioner took consent and provided participants with the baseline questionnaire. Information was supplied to the co-ordinating centre on all eligible patients and outcomes recorded as ‘missed’ , ‘recruited’ , ‘refused’ , with reasons given for ‘missed’ and ‘refused’. Demographic details were taken with consent from those who declined entry to the study. All subsequent questionnaires were sent directly from the co-ordinating centre. Following the pilot the reduced level of consent was introduced to enable the collection of clinical data about those who found questionnaire completion a burden.

The study protocol and measures have been previously published [9]. The study was sponsored by the University Hospital Southampton NHS Foundation Trust Research and Development Office (R&D number: RHM CAN0737) and ethical approval was given through the Oxfordshire REC B National Research Ethics Committee (REC reference number: 10/H0605/31).

### Set up: Identify and contact

The first process in Patterson et al’s [8] framework is to identify and make contact with the various gate keepers which must be negotiated to gain access to the target population. They suggest the most effective way to do this is by face to face contact and the use of multiple communication channels; with the maintenance of good relationships in these early tasks being key. These relationships were not already in place prior to this study which would have enhanced understanding and communication, so much work was done to create these relationships. As this was a large, national study involving 30 cancer centres, the opportunities for personal and face to face contact were limited, so we selected a method by which centres opted in to the study, rather than being approached. The study was submitted to and adopted by the National Cancer Research Institute Clinical Studies Group for Colorectal Cancer. An invitation was sent via the NCRN to all cancer network research managers to submit an Expression of Interest to participate in the study. The criteria used for selection of participating centres were set to enhance the chance of both rapid recruitment and also recruitment of a high percentage of eligible people. These included: a single point where all patients with colorectal cancer could be identified and screened (this was normally a MDT) and for an identified person to be present to identify every eligible patient; one key clinic prior to surgery where every eligible patient could be approached; no competition with other studies; at least one staff member available for the training day; the ability to recruit at a rate of two to three participants a week; local research governance procedures to be completed within six weeks. Other factors taken into consideration when selecting centres were to cover varied socio-demographic and geographical locations. Selected centres were contacted by phone, including conference calls, and email and careful work done through these communications to set up good relationships. Identification and approach of eligible patients would normally be by dedicated research nurses, supported by the NCRN, although this was not a requirement.

### Alliance: connect and engage

Connecting is described by Patterson et al. [8] as engaging the hearts and minds of the gatekeepers, by establishing common ground, in order to develop a shared goal and for the gatekeeper to become an ally in the recruitment process. As the underlying principles of this piece of research were about recovery and psychosocial wellbeing it was thought common ground was more likely to be established with local nursing staff and these became the prime focus of our engagement. We suggested that it would be appropriate for clinical nurse specialists or lead research nurses to be local Principal Investigators which would give them more authority and a vested interest in promoting and undertaking effective research.

Engagement is interactive and intellectual, depending on research credibility, demonstrated through reasoned and critical discussion of the evidence-based approach and study methods [8]. Once the pilot was complete a training day was held for Principal Investigators and research nurses. Travel and accommodation were paid for recruiting centre staff to attend. This face to face day was essential to establish a trusting relationship between the researchers and staff at the participating centres. The study design, purpose and methods were described, for example, stressing the importance of inviting ALL eligible people to participate in CREW, in order to avoid bias, allowing time for a sharing of knowledge and processes between centres. Studies conducted through the NCRN are typically randomised controlled trials, therefore the understanding of observational cohort studies was limited and education focussed on the need to understand the cohort as a whole. People who had had cancer were involved in all these training days, so that they could share from their perspective why the study was important and to support the research nurses in recruitment issues. The research nurses rehearsed recruitment and other study procedures, which varied slightly at each centre. Two further training days were held as these were found to be the most effective way of enabling engagement. Following the training, a teleconference was conducted with each individual site to initiate the study. Ongoing support from the co-ordinating centre took the form of continued frequent email and telephone communications between the centres and the lead researcher or study administrator throughout the set-up and recruitment period to provide updates and address set-up and recruitment queries promptly.

### Exchange: request and resolve

Once a shared goal is established the researcher frames requests to fit with the goal and resolution is reached with an overt agreement about further contact, which is mutually respectful [8]. Gatekeepers require specific, achievable and realistic requests with clear time boundaries. Participating centres were requested to send weekly updates on recruitment status. In order to clarify and simplify what was required of participating centres, a central administrator provided clear requests for any outstanding information. To maintain engagement, a monthly newsletter was distributed to centres to update them on progress and to share best practice. A website was also developed for the recruiting nurses to share best practice. The third study day was held near the end of recruitment and recruiters were able to feed into the process of data gathering and sharing. Detailed evaluation was gathered at this event on issues around the conduct of the study, difficulties encountered and potential solutions shared. This was also used as a celebration of successful recruitment which enhanced the sense of a shared goal and set the scene for future effective collaborations.

## Results

### Selection of participating sites

**Figure 1:**
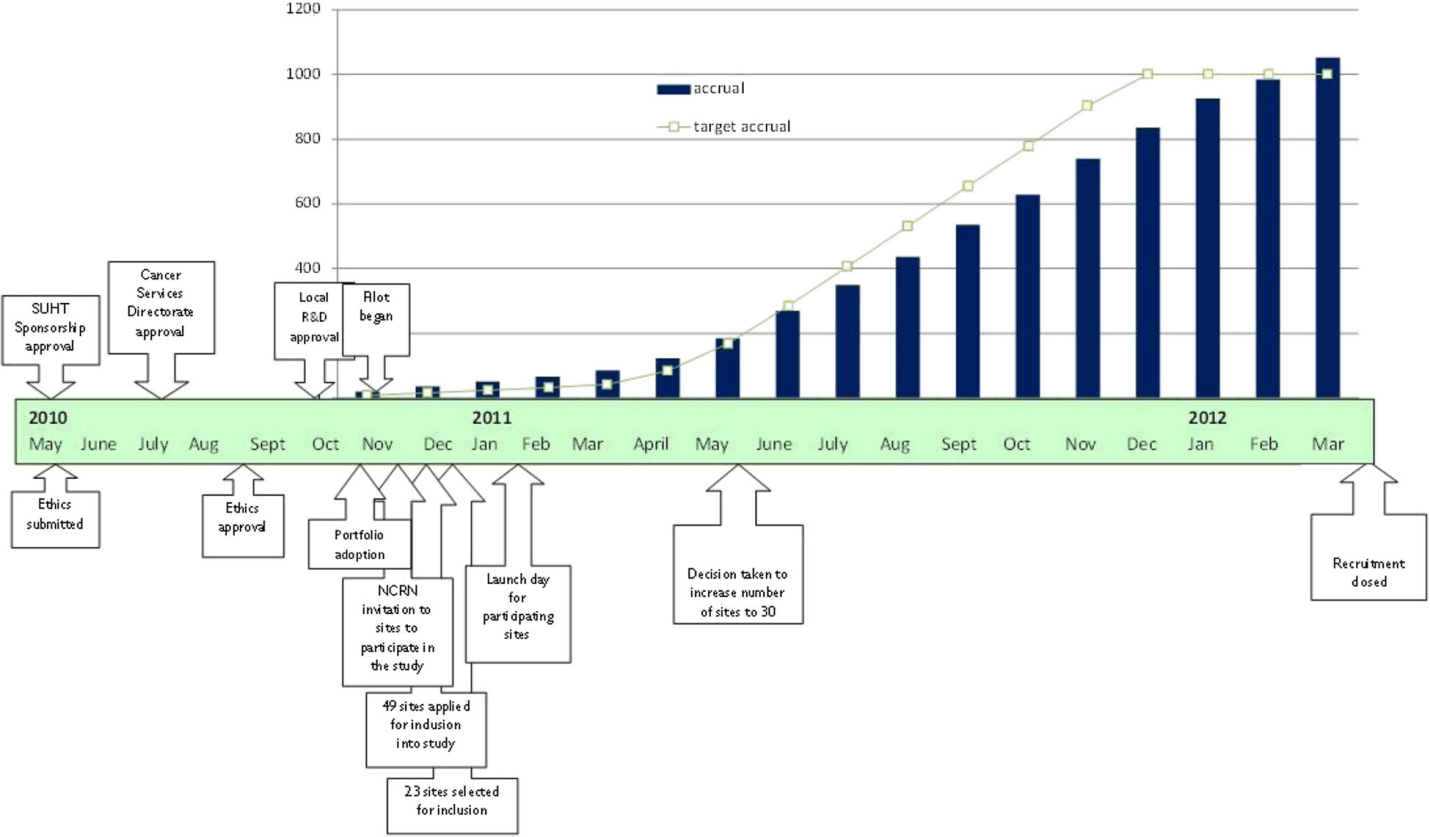
Time taken to conduct study from initial approvals to achieving target recruitment.

**Figure 2:**
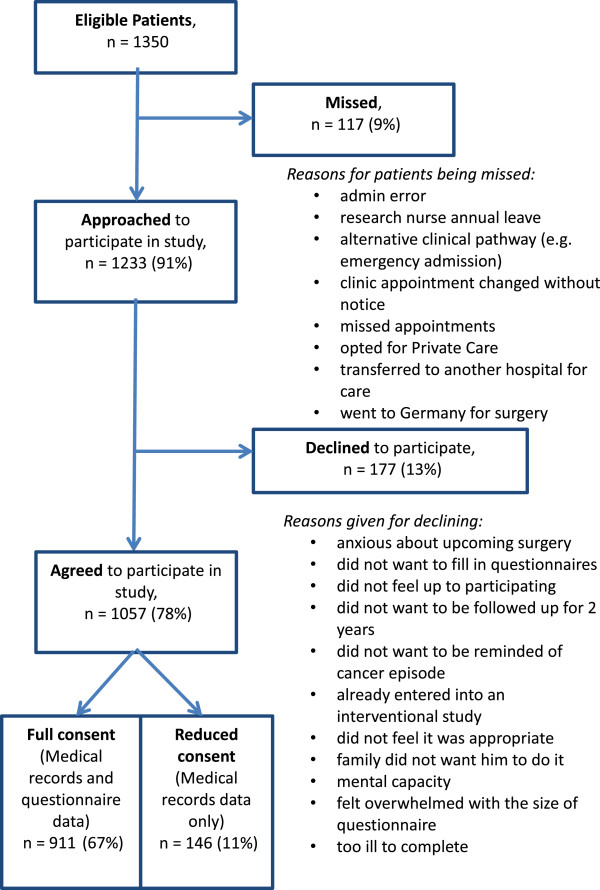
Patient recruitment flow from numbers eligible to participate to numbers consented to study.

After the initial invitation, 49 centres submitted an Expression of Interest through the NCRN, from which 21 plus the pilot site were selected. Several centres in Scotland and Northern Ireland (not in the NCRN) were approached directly and two were selected. All centres met the selection criteria. Evidence of a close working and committed team (surgeons, research nurses and clinical nurse specialists) and a track record of recruitment to previous studies were taken into consideration. Participating centres were informed of selection in December 2010 and expected to start recruiting in February 2011, following the study set-up training day in January. In May 2011, not all centres had begun recruitment and the overall recruitment rate was slower than expected. The decision was therefore taken to increase the number of recruiting centres to ensure recruitment targets were met within the allocated time frame and a further six centres were selected. One of the selected centres failed to screen or recruit eligible patients and was therefore excluded from analysis and study reporting. Ultimately 29 centres actively contributed to recruitment of participants. Time taken to conduct the study from initial approvals to achieving target recruitment is shown in Figure 1. Patient recruitment flow from numbers eligible to participate to numbers consented to study is shown in Figure 2. Each centre has been presented with the opportunity to verify the data in this paper.

### Attendance at training days

The initial study set up training day was attended by 29 people from 17 centres. Six centres failed to send anyone despite prior notice and this being a condition for selection. The other six centres were selected following this initial training day. This training day was useful to engage in mutually respectful exchange of knowledge and enabled us to confirm and consolidate our communication processes with the participating centres. For example, centres indicated that they would like: information on total recruitment to the study and how they compared to other centres; regular newsletters with updates; helpful tips about the study and how to share best practice and that email was the preferred form of communication. All of these were acted upon and put into place. The second training day was attended by 21 people from 16 centres. This day was intended to be a set up training day for the newly selected centres (four out of six attended) but many other centres took the opportunity to be updated on the study and to resolve issues that had arisen during recruitment. Feedback from this day that was acted upon included: building the website for research nurses to share best practice and the preparation of a set of slides for the nurses to present to their MDTs in order to enhance engagement at the local sites. The final day was at the completion of recruitment and was attended by 20 people from 14 centres. The opportunity was taken at this time to discuss what had been difficult about the study processes and to consider how these procedures could be improved in the future.

### Time to achieve recruitment target

The total time from study approval in the leading NHS site to recruitment close across all sites was 23 months. This included six months for sponsorship, ethical approval (following revision), portfolio adoption, local cancer services directorate approval and local R&D approval (Table 1).

**Table 1:** Time (days) taken per centre to commence recruitment, including research approvals, time to screen for eligible patients and time to first recruit (in ascending order according to the total time to recruit)

**Centre**	**Site selection to research approvals submission**	**Research submission to research approval**	**Total time from site selection to research approval**	**Research approval to coordinating centre permission**	**Coordinating centre permission to 1st screening**	**1st screen to 1st patient recruited**	**Research approval to 1st patient recruited**	**Total site selection to 1st recruit**
C	13	35	48	5	0	0	5	53
BB	0	42	42	0	0	17	17	59
B	13	23	36	0	10	15	25	61
D	26	20	46	8	6	7	21	67
E	49	0	49	0	11	12	23	72
F	*Data not available*		63	3	4	4	11	74
G	25	16	41	12	10	12	34	75
H	35	0	35	0	48	0	48	83
I	35	28	63	0	27	0	27	90
J	22	32	54	15	15	7	37	91
K	15	49	64	10	17	0	27	91
L	54	9	63	26	6	0	32	95
M	29	40	69	12	29	1	42	111
N	42	55	97	13	7	2	22	119
O	21	56	77	6	36	5	47	124
P	31	26	57	6	8	55	69	126
Q	35	22	57	3	57	9	69	126
R	48	40	88	37	6	13	56	144
S	*Data not available*		112	0	8	40	48	160
T	*Data not available*		13	0	135	19	154	167
U	62	50	112	0	29	31	60	172
V	0	45	45	49	71	10	130	175
W	145	2	147	0	11	21	32	179
X	34	146	180	5	3	0	8	188
Y	29	89	118	0	61	15	76	194
Z	137	35	172	0	28	6	34	206
AA	28	64	92	0	116	15	131	223
A	21	56	77	4	136	14	154	231
CC	145	2	147	0	129	14	143	290
**Median**	30	35	63	3	15	10	37	124
**Max**	145	146	180	49	136	55	154	290
**Min**	0	0	13	0	0	0	5	53

The median number of days from a site being selected to recruiting the first participant into the study was 124 days (range 53–290) (see Table 1). This included research approval, local set up, screening participants to first participant consented. There is now a national target in place for this to take place within 70 days. In this study only 6 centres completed these processes within 70 days.

The time taken from a centre being informed that they had been selected to obtaining local research approval varied from 13 to 180 days (median 63). Breaking this figure down further, from site selection to research submission ranged from 0 to 145 days (median 30) and from research submission to approval took 0 to 146 days (median 35). Once research approval had been obtained, the time taken to give permission to open the study took from 0 to 49 days (median 3). This delay was due to staff holidays and delays in the coordinating centre being informed that local research approval had been granted. The time taken from the co-ordinating centre giving permission to open the study to the date of first screening by the MDT ranged from 0 to 136 days (median 15) and then from screening to the first patient recruited by a centre was 0 to 55 days (median 10). These data per recruiting centre are given in Table 1. Several centres had long delays from when the study was officially opened at their site to when they started to actively recruit to the study. These were due to staffing issues, such as sick leave and change of staff.

### Recruitment rates

It took 17 recruitment months to reach the final sample of 1056 participants with a mean recruitment rate per centre of 0.71 participants per week (Table 2). Predicted recruitment time was 12 months, plus two months for the pilot. Over the period of time that the participating centres were recruiting, 1350 people were screened as being eligible for the study. Ninety one per cent of these were approached about participating in the study (9% missed); of whom 910 (67% of total eligible) gave full consent to study participation. A further 146 (11%) gave consent at the reduced level for their routinely collected data to be used, without questionnaire completion, and 177 (13%) declined to participate (see Table 2). Following surgery, 34 participants who had given full consent were found to be ineligible due to Duke’s stage, metastases or cancer misdiagnosis. These have been removed from analysis giving a total cohort of 1022. Basic demographic data, including gender, age, ethnicity, relationship status, employment status and postcode (as a measure of social deprivation), were collected on decliners. The participants recruited to the study were significantly younger and more likely to be in employment than those who declined or gave reduced consent. However, large numbers of older people gave full consent to the study, with 48% of the total sample being over 70 years of age and 15% over 80. Older people were more likely to give reduced consent, which ensured that we were able to collect data on them. Overall 91% of people aged under 70 and 86% people aged over 70 gave either full or reduced consent to the study.

**Table 2:** Rate of recruitment per centre, including expected rate, missed, declined and total recruited (in ascending order according to Recruitment Index)

**Centre**	**Number of weeks recruiting**	Expected recruitment per week*	Expected total recruitment figure**	**Total eligible patients**	**Missed**	**Declined**	**Reduced consent**	**Full consent**	**Totals recruited**	**% recruited (total recruited/total eligible)**	**Rate per week**	Recruitment index***	**Attended initial set up day?**	**Total number of nurses attending study days**
BB	80	2	160	217	19	9	24	165	189	87%	2.36	2.06	Yes	4
B	56	3	95	130	6	15	22	87	109	84%	1.95	1.64	Yes	5
L	54	2	104	115	3	10	11	91	102	89%	1.89	1.68	Yes	3
J	56	2	111	94	0	4	7	83	90	96%	1.61	1.55	Yes	5
K	55	1	55	53	1	1	12	39	51	96%	0.93	0.89	Late start	2
CC	26	5	130	25	1	0	5	19	24	96%	0.92	0.88	Yes	3
M	52	2	104	68	7	7	3	51	54	81%	1.06	0.86	Yes	7
W	43	1	43	39	1	2	10	26	36	92%	0.84	0.77	Yes	3
C	60	2	120	52	2	5	12	33	45	87%	0.75	0.65	Yes	3
Z	15	2	31	11	0	1	0	10	10	91%	0.67	0.61	Late start	1
O	51	2	101	57	9	8	8	32	40	70%	0.78	0.55	Yes	3
X	44	3	131	32	6	1	0	25	25	78%	0.57	0.44	Yes	1
Y	42	1	42	28	1	5	2	20	22	79%	0.52	0.41	Yes	4
Q	52	2	111	38	1	9	1	27	28	74%	0.54	0.40	Yes	4
N	46	2	91	41	0	14	4	23	27	66%	0.59	0.39	Late start	2
D	59	2	118	49	3	13	0	33	33	67%	0.56	0.38	Yes	1
A	37	2	73	18	0	2	2	14	16	89%	0.43	0.38	Yes	5
F	38	2	77	47	11	10	7	19	26	55%	0.68	0.37	Late start	2
G	38	2	75	22	3	2	5	12	17	77%	0.45	0.35	No	0
I	55	3	167	52	12	11	5	24	29	56%	0.53	0.30	Yes	4
H	56	1	47	41	4	11	1	25	26	63%	0.46	0.29	Yes	2
V	47	4	187	17	3	3	0	11	11	65%	0.23	0.15	No	1
U	47	2	44	30	7	9	0	14	14	47%	0.30	0.14	Yes	3
E	38	2	76	37	5	20	4	8	12	32%	0.32	0.10	Late start	1
AA	38	2	109	11	2	3	1	5	6	55%	0.16	0.09	No	0
S	35	2	69	3	0	0	0	3	3	100%	0.09	0.09	No	0
R	52	2	103	12	4	1	0	7	7	58%	0.13	0.08	No	0
T	22	2	108	1	0	0	0	1	1	100%	0.05	0.05	No	0
P	34	1	34	10	5	2	0	3	3	30%	0.09	0.03	Late start	1
			**TOTAL**	**1350**	**116**	**178**	**146**	**910**	**1056**	**78**%				70
					**9**%	**13**%	**11**%	**67**%	**78**%					

*As declared by centre on Expression of Interest and/or Contract (rounded down).

**Number of weeks recruiting x Expected recruitment per week.

***Recruitment Index = mean number recruited per week x percentage of eligible people actually recruited.

The most effective recruiting centres were those who not only recruited large numbers of people into the study, but also recruited a high percentage of the eligible people, thus ensuring a representative sample. A Recruitment Index has been proposed by Rojavin [10] which provides a measure of the number of days required for an average study site in a multicentre study to recruit one analysable participant. However, this measure does not take into account frequency of screening failure which was important for this study. We therefore calculated a recruitment index (RI) for each individual centre as a factor of the rate of recruitment per centre (the mean number recruited per week) and the percentage of the people eligible for the study who were actually recruited (Table 2).

### Challenges and strategies to enhance study set up and recruitment

Data from the final evaluation day suggested three main areas which affected effective study set up and ongoing recruitment. These were engagement, ongoing communication and adequate resourcing.

The first phase of set up [8] involved identifying and engaging the relevant gate keepers. While we were able to identify initial gate keepers through the networks, it became clear that there were a number of gatekeepers in each organisation and it was important to communicate with all of these gatekeepers. In this study, this included research nurses, colorectal cancer clinical nurse specialists, colorectal cancer surgeons and administrators. It could not be assumed that there was good internal communication. One centre reported that the research nurse was the last to know about the study. Some nurses stated that they found it difficult to complete the Expression of Interest (EOI) form. Many of the issues that resulted in poor recruitment were when centres did not keep to the processes outlined in the EOI form, particularly having a single MDT meeting and a single pre-assessment clinic to identity patients. This could have been reduced if centres had received more help to complete this initial EOI form and clarification reached about whether these processes were possible.

Education of and relationship building with participating site research teams was essential to enable rapid study set-up. The training days and weekly communications were the most effective way that this was achieved. However, connection and engagement with internal gatekeepers was repeatedly raised as an issue by the research nurses. Once their commitment was secured, they needed support from the team at the co-ordinating centre to engage and secure commitment from other MDT members, particularly the clinical nurse specialists (CNS) and surgeons. They also valued the support, where it was available to them, of administrative staff. Research nurses stated that they did not have sufficient specialist knowledge to identify eligible patients and so were dependent on CNSs. They suggested that the CNSs should have been formally invited to the initial study training day and that attendance at the initial training day was a compulsory precondition for acceptance as a participating centre. The most effective recruiting centres were those with which we had made most effective connections and engagement, particularly where we had made relationships with both the research nurses and the CNSs. The level of engagement can be demonstrated by the number of staff that attended our study days. The total number of staff attending any of our study days was positively correlated (*r* = 0.59) with the Recruitment Index achieved by that centre. Furthermore, of the five poorest performers, none attended the initial study day and the top 15 performers all attended the initial study day.

Nurses appreciated the opportunity to be Principal Investigators for their site and the fact that this study was about recovery of wellbeing after cancer meant that shared values and goals were quickly established. Research nurses also appreciated the opportunity to have their views heard; one said *‘Thank you for valuing our input’*. This suggested that we were able to achieve a mutually respectful relationship.

The exchange phase of the study was also very important for effective recruitment and required ongoing communication. The weekly communication included a summary of all data collected, including all individual study sites and summary data. We also developed a newsletter for the participating sites which included ‘frequently asked questions’ and tips from those centres that had good recruitment rates. There was a website for the research nurses with resources available, such as a slide presentation on the study for research nurses to present to their MDT members. Positive feedback from the final training day included comments about easy access to the co-ordinating centre, rapid feedback to queries, accessibility to the Chief Investigator when needed, the value of regular feedback, on recruitment rates, the newsletters and the training days. Difficulties were a lack of understanding about the nature of cohort studies and there was a suggestion that more training days would have been helpful.

Resourcing was an issue at all stages of the process. Staff absences were a problem; if any key staff member was absent, this could cause delay to both the set-up and the recruitment process. There was frequently no cover provided for research nurse absence, which has a potential impact of being able to recruit a high percentage of eligible patients, and research nurses were often overstretched, needing to cover many different studies, attending clinics which could be running in parallel or travelling between different hospital sites (Table 3).

**Table 3:** Reasons offered for delays in set up and running of the study

**Problem reported**	**Number of centres reporting this problem**
Sick leave/annual leave	8
Research nurse overstretched by having to cover many different studies, clinics or a number of hospitals	5
No single point of access for potential participants (e.g. patients attending for assessment prior to surgery could attend any day of the week)	3
Competing studies. Priority given to clinical trials	2
PI &/or research nurse unable to engage the rest of the MDT	2
Research nurse said patients did not want to join study	2
Research nurse leaving	2
MDT processes changed since submitting EOI	1
Contract issues	1
PI did not have GCP training	1

Note. PI Principal Investigator, MDT Multidisciplinary team, EOI Expression of Interest, GCP Good Clinical Practice.

Only one centre, which was the pilot site, achieved the expected recruitment rates. There are a number of reasons for this, most of which are organisational issues. Clinicians generally overestimated the number of eligible patients attending their centres. This could have been addressed in part by a stronger focus on the eligibility criteria for the study or by asking the sites to monitor eligibility prior to study entry. Despite initially checking that there were no conflicting studies, some sites subsequently reported competing studies and clinical staff were reluctant to ask patients to participate in more than one study. Drug studies were likely to be given priority over epidemiological studies.

## Discussion and conclusions

This study was successful in recruiting a high percentage of eligible patients, despite the fact that they had recently been diagnosed with colorectal cancer. Furthermore, the period of recruitment was only three months longer than predicted, despite not having an established relationship with the recruiting centres prior to the commencement of the study. The use of a reduced level of consent enabled the inclusion of a high percentage of older people who are normally underrepresented in cancer studies.

The use of a theoretical framework helped to enhance processes of engagement and relationship building, which had a beneficial impact on effective recruitment. Key differences in the way that this study was run included careful pre-screening and selection of participating centres. Engagement was initiated by inviting centres to submit an application to join the study, and to commit to a number of predetermined selection criteria. This is not a routine method used to select participating centres in UK trials. To improve this process, further preconditions could be set, such as minimum recruitment index as well as predicted recruitment rate. Attendance at the study set up day was important for communication and education and should also be a requirement for selection. Enabling nurses at the recruitment centres to be local Principal Investigator was unusual and enhanced the sense of personal connection and engagement with the research, as nurses were largely responsible for recruitment. Ongoing communication was also important and the frequency of communication and training was greater than in other UK portfolio studies.

The detailed level of data reported here could be used for centres to measure performance against, including time to obtain research governance approvals and recruitment index. The study as a whole recruited at a median of 0.59 per centre per week (over two per month) and a recruitment index of 0.39 (mean number recruited per week x % eligible). These figures could be set as targets and be part of the contractual agreement prior to study commencement.

Recruitment to this study took place in 2010 (including pilot) until 2012 and successfully recruited 1056 participants in 17 months. It is noticeable how long it took, and the degree of variability amongst, study centres to process their research governance procedures, with a range of 13 to 180 days (median 63). All the participating sites claimed that they could fulfil local research procedures within six weeks, but only four centres achieved this. Since this study has been completed, new targets have been introduced designed to streamline the research governance procedures at local centres participating in UK multi-centre studies, and this aspect of study set up should now be greatly improved.

The large variations in time to commence recruitment following permission to start appeared to be largely due to resource and staffing issues. Full support needs to be given to the recruiting sites to ensure they understand study procedures. Clearly, management of the local research portfolio is a complex issue and the inability to cover staff absence has major implications for study effectiveness.

There were also large variations between centres in their effectiveness at recruiting a high percentage of eligible patients. Addressing the issues at the root of this variation may enhance recruitment to other studies. Identification and screening of eligible patients is complex, but could be enhanced by electronic systems. For high quality research it is important not just to recruit high numbers, but that a high percentage of eligible patients are recruited, in order to reduce bias and ensure representation of the whole population under study. Recruitment of potentially vulnerable patients into studies near to primary diagnosis and undergoing invasive treatment for cancer requires well trained and supported staff, dedicated to this work. This is particularly important to reach those who are typically underrepresented in research, such as the frail, elderly or with low literacy, and adequate time and support is needed to enable these people to make appropriate decisions about research. Clinical systems in many areas, particularly large cancer centres, are not designed to facilitate this. In order for research to be effective and of high quality, research must be seen as integral to and high priority for multidisciplinary working, with commitment from all team members, and adequate resourcing of research nurses.

Working with the NCRN from the outset and inviting sites from throughout the UK to submit an Expression of Interest (EOI) was a valuable way to commence recruitment as speedily as possible. One to one discussions about the EOI could have enhanced the selection process and initiated development of relationship building with participating centre. Development of good working relationships with study sites was essential to the success of this study, built on frequent and regular contact by phone, email and face to face. Adequate resourcing of the coordinating centre was crucial to allow this.

## Competing interests

The authors declare that they have no competing interests.

## Authors’ contributions

CF and JC conceptualised the project, and obtained study funding; CF is the Chief Investigator, and DF, AR, JAH, PS, JC, JW are co-investigators. DF developed the study protocol. DF and KCS prepared the manuscript. DF oversaw the day-to-day running of the study during the recruitment phase working with IO, KCS, CM and MB. All authors read and approved the final manuscript.

## Pre-publication history

The pre-publication history for this paper can be accessed here:

http://www.biomedcentral.com/1471-2288/13/153/prepub
